# An Association between BK Virus Replication in Bone Marrow and Cytopenia in Kidney-Transplant Recipients

**DOI:** 10.1155/2014/252914

**Published:** 2014-04-29

**Authors:** Emilie Pambrun, Catherine Mengelle, Geneviève Fillola, Patrick Laharrague, Laure Esposito, Isabelle Cardeau-Desangles, Arnaud Del Bello, Jacques Izopet, Lionel Rostaing, Nassim Kamar

**Affiliations:** ^1^Department of Nephrology and Organ Transplantation, CHU Rangueil, TSA 50032, 31059 Toulouse, France; ^2^Department of Virology, CHU Purpan, 31059 Toulouse, France; ^3^Laboratory of Haematology, CHU Rangueil, 31059 Toulouse, France; ^4^Université Paul Sabatier, 31000 Toulouse, France; ^5^INSERM U1043, IFR–BMT, CHU Purpan, 31059 Toulouse, France

## Abstract

The human polyomavirus BK (BKV) is associated with severe complications, such as ureteric stenosis and polyomavirus-associated nephropathy (PVAN), which often occur in kidney-transplant patients. However, it is unknown if BKV can replicate within bone marrow. The aim of this study was to search for BKV replication within the bone marrow of kidney-transplant patients presenting with a hematological disorder. Seventy-two kidney-transplant patients underwent bone-marrow aspiration for cytopenia. At least one virus was detected in the bone marrow of 25/72 patients (35%), that is, parvovirus B19 alone (*n* = 8), parvovirus plus Epstein-Barr virus (EBV) (*n* = 3), cytomegalovirus (*n* = 4), EBV (*n* = 2), BKV alone (*n* = 7), and BKV plus EBV (*n* = 1). Three of the eight patients who had BKV replication within the bone marrow had no detectable BKV replication in the blood. Neutropenia was observed in all patients with BKV replication in the bone marrow, and blockade of granulocyte maturation was observed. Hematological disorders disappeared in all patients after doses of immunosuppressants were reduced. In conclusion, an association between BKV replication in bone marrow and hematological disorders, especially neutropenia, was observed. Further studies are needed to confirm these findings.

## 1. Introduction

Hematological abnormalities, that is, anemia, leucopenia, and thrombocytopenia, are commonly observed in kidney-transplant patients [1, 2]. Apart from anemia caused by impaired kidney function, most cases of cytopenia are related to viral infections or to bone-marrow toxicity caused by drugs used at posttransplantation [1–3]. In cases of cytopenia, viral infection is usually ruled out by searching for the viral genome in blood or in blood-marrow aspirates. Parvovirus B19 infection is a classic cause of anemia [4], and cytomegalovirus (CMV) is well known to suppress bone-marrow function [5].

Patients who present with severe cytopenia, and in whom bacterial, viral, and fungal infections have been ruled out, should be assessed for possible toxic causes for these hematology abnormalities. Indeed, several drugs that are frequently used after transplantation can suppress bone-marrow activity; these include the mycophenolates, azathioprine, the mammalian target of rapamycin inhibitors, (val) ganciclovir, and cotrimoxazole [1–3]. This toxicity can lead to immunosuppressants being discontinued and, thus, an increased risk of acute rejection [6], or the withdrawal of prophylactic drugs, which increases the risk of infections [3].

The human polyomavirus, BKV, is associated with severe complications, such as ureteric stenosis and polyomavirus-associated nephropathy (PVAN), which often occurs in kidney-transplant patients, and polyomavirus-associated hemorrhagic cystitis, which preferentially affects patients who have received an allogeneic hematopoietic stem-cell transplant [7]. BKV replicates in many cell types, particularly in peripheral blood mononuclear cells and in epithelial urinary cells [7, 8]. It has been also demonstrated that BKV has a tropism to vascular endothelial cells [9]. A case of BKV-related hemophagocytic syndrome has been observed in a kidney-transplant patient [10]. Finally, BKV replication has been observed in the bone marrow and in the blood in a kidney-transplant patient [11]. The aim of our study was to search for BKV replication within the bone marrow of kidney-transplant patients presenting with a hematological disorder.

## 2. Patients and Methods

In our institution, all kidney-transplant patients are screened for BKV in the blood at months 1, 3, 6, and 12 after transplantation and yearly thereafter, as well as each time they present with kidney function impairment. Between January 2007 and May 2012, all kidney-transplant patients who presented for cytopenia were screened prospectively for BK virus in the blood and bone marrow. The study was approved by Toulouse University Institutional Review Board. Hence, 72 kidney-transplant patients underwent bone-marrow aspiration for cytopenia. Of these, seven patients had a combined transplantation: heart and kidney (*n* = 1), liver and kidney (*n* = 2), and kidney and pancreas (*n* = 4). During the same period, five patients underwent bone-marrow aspiration for a reason other than a hematological disorder, that is, suspected posttransplant lymphoproliferative disease (*n* = 4) and monoclonal gammopathy of undetermined significance (*n* = 1).

Aspiration of bone marrow was performed when hemoglobin level had decreased to <11 g/dL, and/or neutrophil count was <1000/mm^3^ and/or platelet count was <120,000/mm^3^. Cytological analyses were performed on each bone-marrow aspirate. In addition to BKV, nuclear-acid tests for classical viruses usually observed in kidney-transplant patients, that is, CMV, EBV, and parvovirus B19, were conducted on the bone-marrow aspirate fluid and on peripheral blood samples.

### 2.1. Virological Analyses

Bone-marrow and whole blood samples were collected in tubes with potassium EDTA. Nucleic acids were extracted from samples with the MagNA Pure 96 instrument using the MagNA Pure 96 DNA and viral NA small volume kit (Roche Diagnostics, Meylan, France) according to the manufacturer's instructions (extracted volume: 200 *μ*L, elution volume: 100 *μ*L). The detection limit for BKV was 500 copies/mL.

CMV [12] and BKV [13] were detected using real-time PCRs on a LightCycler. EBV DNA was detected using the quantitative Epstein-Barr virus real-time PCR (Diagenode, Liège, Belgium). Parvovirus B19 was assessed using the RealStar Parvovirus B19 PCR Kit (altona Diagnostics Hamburg, Germany). The detection limit for CMV, EBV, BKV, and parvovirus B19 was 500 copies/mL.

### 2.2. Statistical Analyses

Reported values represent the mean (±SD) or medians (ranges). Proportions were compared using Fisher's exact test. Quantitative variables were compared using the Mann–Whitney nonparametric test or Student's *t*-test. A *P* value of <0.05 was considered statistically significant.

## 3. Results

### 3.1. Characteristics of Patients with Hematological Disorders (Table 1)

Thirty-two of the 72 patients (44%) had experienced an acute rejection episode before presenting with a hematological abnormality, that is, thirteen patients presented with steroid-sensitive acute rejection, which was treated with steroid pluses; nine patients developed steroid-resistant acute rejection, which was treated with rabbit antithymocytes globulins; and ten patients experienced an antibody-mediated rejection, which was treated with plasma exchanges and rituximab (Table 1). Two of these latter ten patients also received intravenous immunoglobulins. Three additional patients received rituximab for relapsed focal segmental glomerulosclerosis (*n* = 2) or for relapsed membranoproliferative glomerulonephritis (*n* = 1).

Seventeen patients had previously presented with CMV replication (24%). Five patients presented after transplantation with at least one occurrence of BKV replication (7%), and two of these five had developed PVAN (2.7%).

### 3.2. Findings from Bone-Marrow Aspirates

#### 3.2.1. Patients with a Hematological Disorder

Aspiration of bone marrow was performed for isolated neutropenia in 24 patients (34%), pancytopenia in 23 patients (32%), bicytopenia in 13 patients (18%), isolated anemia in 8 patients (11%), and isolated thrombocytopenia in 4 patients (5%). The median time between kidney transplantation and aspiration of bone marrow was 10.5 (range: 1–433) months. Median neutrophil and platelet counts in the blood were 800 (range: 20–8851)/mm^3^ and 135,000 (range: 11,000–529,000)/mm^3^, respectively. Hemoglobin level was 11 (range: 6.3–16.5) g/dL.

At least one virus was detected in the bone marrow of 25 of the 72 patients (35%). Parvovirus B19 alone was detected in eight patients, parvovirus plus EBV in three patients, CMV alone was detected in four patients, EBV alone in two patients, BKV alone in seven patients, and BKV plus EBV was detected in one patient. Concomitantly, a virus was detected in the blood of several of these patients (Table 2).

For 38 of the 72 patients, bone-marrow analyses revealed the presence of nonspecific dysmyelopoiesis and no viruses were detected. However, 12 of these 38 patients had detectable viruses in their blood. In nine other patients, bone-marrow aspirates revealed thrombotic microangiopathy (*n* = 3), tuberculosis (*n* = 2), myeloid acute leukemia (*n* = 1), or hemophagocytic syndrome (*n* = 3). In four of these nine patients, a virus was detected in the peripheral blood (Table 2).

#### 3.2.2. Patients without a Hematological Disorder

Among the five patients who underwent bone-marrow aspiration for a reason other than cytopenia, no virus was detected in bone-marrow aspirates or in the blood.

### 3.3. Characteristics of Patients Who Had a Hematological Disorder and BKV Replication within the Bone Marrow

BKV replication in bone marrow was found in 11% (8/72) of patients. Their characteristics are presented in Tables 3, 4, and 5. The median time between kidney transplantation and aspiration of bone marrow was 13.5 (range: 4.5–46.5) months.

#### 3.3.1. Bone-Marrow Analyses

Aspiration of bone marrow was performed for isolated neutropenia (*n* = 3), pancytopenia (*n* = 2), neutropenia plus thrombocytopenia (*n* = 2), and neutropenia plus anemia (*n* = 1). Neutropenia was observed in all patients who had BKV replication within the bone marrow (Table 3). Microscopic analyses of the bone marrow revealed hypocellularity, which mainly affected the myelopoietic line. Granulocyte maturation was blocked in all patients. In addition, polymorph lymphocyte proliferation was observed in one patient, and one patient had features of hemophagocytic syndrome. Anemia can be in part attributed to impaired kidney function (Table 3).

#### 3.3.2. Immunosuppressive Therapy Given to Recipients with BKV Replication within Bone Marrow

All patients received an induction therapy of polyclonal antibodies (*n* = 3) or anti-interleukin-2 receptor (IL2R) blockers (*n* = 5) (Table 4). Four patients had experienced an acute-rejection episode before the hematological disorder: two had steroid-sensitive acute rejection, which was treated with steroid pulses (Patients 1 and 6), one patient had a combined cellular and humoral rejection, which required steroid pulses, plasma exchanges, and rituximab (Patient 7), and one patient, who had received anti-IL2R blockers as an induction therapy, experienced steroid-resistant acute rejection, which was treated with steroid pulses and polyclonal antibodies (Patient 5). One of the two patients that experienced steroid-sensitive acute rejection presented later with relapsed membranoproliferative glomerulonephritis, which was treated with plasma exchange and rituximab (Patient 6). Hence, overall, four patients received polyclonal antibodies and two patients received rituximab therapy.

At the time of bone-marrow aspiration, six patients were receiving calcineurin-inhibitor- (CNI-) based immunosuppression, one patient was receiving sirolimus, and one was receiving belatacept. All patients were also receiving mycophenolic acid (MPA), except for one, who received leflunomide for PVAN. All patients were given steroids.

#### 3.3.3. Concomitant Viral Replication

Seven patients had isolated BKV replication within the bone marrow. The median BKV viral load in bone marrow was 3.02 (range: 2.74–3.24) log_10_ copies/mL. Five of these seven patients had concomitant BKV replication in the blood (3.2 ± 0.96 log_10_ copies/mL) whereas the other two had no BKV replication in peripheral blood. An eighth patient had concomitant BKV (3.01 log_10_ copies/mL) and EBV replication in the bone marrow and isolated EBV replication in the blood. Hence, overall, three patients had detectable BKV replication in the bone marrow (2.94, 3.01, and 3.24 log_10_ copies/mL) but BKV replication was not detected in the blood and in the urine that were assessed twice at one week interval after bone marrow aspiration. None of these 3 patients had a past history of BK virus replication in blood and none of them had a detectable BK virus replication in the blood until April 2014.

Two of the patients with a hematological disorder and BKV replication within the bone marrow had a history of BKV positive viremia, but only one patient developed PVAN that was ongoing.

Of the total 72 patients, nine patients had positive BKV viremia. Five of these also had BKV replication in the bone marrow (whole blood viral load: 3.2 ± 0.96 log copies/mL), whereas the other four did not (whole blood viral load: 3.85 ± 1.53 log copies/mL; *p* = ns).

#### 3.3.4. Treatments and Outcomes

For patients receiving CNIs and MPA (*n* = 5), the MPA was either stopped (*n* = 3, patients 4, 6 and 7) or the dose was decreased by 50% (*n* = 2, patients 5 and 8) without any modification to CNI dose. All five patients received granulocyte colony-stimulating factors (GSF). Patient 4 was also given intravenous immunoglobulins (total dose of 2 g/kg). Patient 6, who had features of hemophagocytic syndrome, was given intravenous immunoglobulins (total dose of 2 g/kg) and steroid pulses (5 mg/kg for 3 days). In the patient who received tacrolimus plus leflunomide (Patient 2), tacrolimus dose was decreased and GSF was given. In the patient who received sirolimus plus MPA (Patient 3), MPA dose was decreased by 50%. Patient 1, who was receiving belatacept plus MPA, was also treated with GSFs.

Hematological disorders disappeared in all patients within 3 to 10 days. No relapse in hematological disorders was observed in all patients but one (Patient 1). No bone-marrow aspirates were performed thereafter, except patient 1. Patient 1, who was receiving belatacept plus MPA, and who was treated with GSFs, presented with severe neutropenia at 3 years after the first episode. BKV replication was again detected in the bone marrow but was still undetectable in the blood. At that time, MPA was withdrawn for 15 days and thereafter sirolimus was introduced instead of the MPA. He did not undergo a control bone marrow aspiration and he did not present any cytopenia episode afterwards.

Of the five patients who had concomitant BKV replication within the blood and bone marrow, one patient had a history of PVAN, which evolved to end-stage kidney disease a few months later. Another patient remained viremic. The other three patients were cleared of the virus at 4, 5, and 57 months after the hematological disorder.

### 3.4. Factors Associated with BKV Replication in the Bone Marrow of Patients with a Hematological Disorder

We searched for the predictive factors for BKV replication in the bone marrow. The proportion of patients having a BKV replication in the blood was significantly higher in the group of patients having concomitantly a BKV replication within bone marrow compared to the group without BKV replication within the bone marrow. Kidney function was worse in patients with BKV replication within the bone marrow (Table 5). Because of the small number of patients having a BKV replication within the bone marrow, no multivariate analysis was performed.

## 4. Discussion

After kidney transplantation, BKV is responsible for some well-known complications, mainly PVAN and, more rarely, ureteral stenosis [7, 8]. However, BK replication within the bone marrow that induces hematological disorders is an unknown possible complication. In our study, we searched for BKV replication in the bone marrow of kidney-transplant patients with a severe hematological disorder. Interestingly, we found that BKV replication was detected in the bone marrow, mainly in patients who had neutropenia, in the presence or not of concomitant BKV replication in the blood. However, we did not identify any predictive factors for BKV replication in bone marrow.

To the best of our knowledge, only one case of BKV replication has been reported: this was in a 17-year-old kidney-transplant patient who was treated for PVAN and presented with severe pancytopenia [11]. BKV replication was detected in the blood and bone marrow at very high viral loads [11]. An allograft nephrectomy and stopping immunosuppression therapy improved the hematological parameters. The authors of this report suggested that BKV may have been responsible for the hematological disorders [11].

Interestingly, among hematological patients presenting with severe neutropenia, with or without fever, BKV was the most common virus detected in the blood [14]. It was detected in 18 out of 158 patients (11.4%). In 13 of these 18 patients, BKV was the sole virus detected in the blood [14]. None of the patients had symptoms of a classical disease associated with BKV [14]. Unfortunately, BKV was not looked for in the bone marrow.

In our study, BKV replication was detected in the blood of nine patients, but only five had BKV replication within the bone marrow. Interestingly, BKV replication was also detected in the bone marrow of three additional patients who had no detectable BKV replication in the blood. Hence, BKV replication was detected in eight of our 72 patients (11.1%). Surprisingly, BKV viral loads in the blood were not as high as it is usually observed in kidney-transplant patients with persistent BKV replication and those with PVAN. Finally, only one patient had EBV replication in the blood and bone marrow in addition to BKV replication in the bone marrow.

The occurrence of three patients with detectable BKV replication within the bone marrow and not in the blood suggests that BKV can replicate in bone marrow and that its detection is not related to blood contamination. In addition, patients who had undergone bone-marrow aspiration for a reason other than anemia, neutropenia, or thrombocytopenia had no detectable BKV replication within the bone marrow. Hence, in addition to its replication in peripheral blood mononuclear cells, epithelial cells, and its tropism to endothelial cells, BKV can replicate within the bone marrow.

In the present study, neutropenia was the most common hematological abnormality, and hypocellularity of the myelopoietic line and blockade of granulocyte maturation were the most common lesions observed in bone-marrow analyses. The mechanism by which BKV can induce bone-marrow suppression is unknown. One can speculate that, similar to other viruses (such as cytomegalovirus), BKV may alter accessory cell function by inducing the production of inhibitory cytokines, it may perturb stromal cell function, resulting in decreased production of hematopoietic factors, it may alter cell-surface adhesion molecule expression, or BKV may directly infect hematopoietic stem-cells or progenitor cells [5]. In order to establish a persistent or lytic infection and cause disease, BKV must be internalized into a host cell type that is permissive to infection. After binding its receptor, BKV must enter the cell and successfully traffic through the cytoplasm toward the nucleus, where the uncoated viral genome can utilize the cellular machinery for transcription and its genome replication. Hence, one can speculate that BKV can infect granulocyte progenitors. Nevertheless, there is no* in vitro* data to support this hypothesis.

Our data suggest that in cases of hematological disorders, in addition to classical viruses that are well known to induce medullar suppression, BKV should be searched for in-bone marrow. If it is detected, a reduced dosage of immunosuppressives can be proposed. In kidney-transplant patients, reducing immunosuppression is considered the first-line therapy to treat BKV replication in the serum and PVAN [8].

In the present study, the majority of patients had their immunosuppressive therapy reduced, which, in addition to granulocyte colony-stimulating factors, improved the patients' hematological parameters. Interestingly, the only patient who was treated with GSF without any modification to his immunosuppressive regimen relapsed 3 years later, and BKV was again detected in the bone marrow. However, we cannot claim that the reduced immunosuppression allowed BKV clearance as the reduction of mycophenolic acid may have decreased the medullar toxicity and, thus, allowed improvement of his hematological disorder. Finally, clinicians should be careful in the reduction of immunosuppression because of the increased risk of acute rejection. Larger studies are needed to understand the causes, consequences, and management of BK virus replication in the bone marrow in kidney transplant recipients.

Our study has several limitations. Other viruses, such as HHV6, were not looked for in the bone marrow. We did not perform a second bone-marrow aspirate to verify that BKV became undetectable after the hematological disorder had been resolved. We considered it unreasonable to propose a bone-marrow aspirate in the absence of a hematological abnormality.

In conclusion, an association between BKV replication in bone marrow and hematological disorders, especially neutropenia, was observed. Further studies are required to confirm these findings.

## Figures and Tables

**Table 1:** Patients' characteristics and bone-marrow aspirates.

Variables	Total patients: *n* = 72
Male gender (%)	40 (56%)
Age at bone-marrow aspiration (years)	57 ± 12
First kidney transplantation (%)	61 (85%)
Diabetes mellitus (%)	13 (18%)
Serum-creatinine level (µmol/L)	212 ± 134
eMDRD GFR (mL/min)	35 ± 21
Immunosuppressive therapy	
Induction therapy (%)	57 (79%)
RATG/anti-IL2R induction therapy (%)	18 (25%)/39 (54%)
RATG for induction or rejection therapy (%)	22 (31%)
Rituximab (%)	13 (18%)
Calcineurin inhibitors (%)	63 (88%)
Cyclosporine A/tacrolimus (%)	51 (71%)/12 (17%)
mTOR inhibitors (%)	4 (5%)
Belatacept (%)	5 (7%)
Mycophenolic acid (%)	60 (83%)
Steroids (%)	72 (100%)
History of acute rejection	
Steroid-sensitive acute-rejection episodes (%)	13 (18%)
Steroid-resistant acute-rejection episodes (%)	9 (12.5%)
Antibody-mediated rejection episodes (%)	10 (14%)
History of viral replication	
Positive BK viremia	5 (7%)
PVAN	2 (3%)
CMV replication	17 (24%)

RATG: rabbit anti-thymocyte globulins; anti-IL2R: anti-interleukin-2 receptors; mTOR: mammalian target of rapamycin; PVAN: polyomavirus-associated nephropathy; CMV: cytomegalovirus.

**Table 2:** Viral replication in bone marrow and blood from kidney-transplant patients with a hematological disorder.

	72 bone-marrow aspirates							
Bone marrow	Parvovirus B19 (*n* = 8)	Parvovirus B19 + EBV (*n* = 3)	CMV (*n* = 4)	EBV (*n* = 2)	BKV (*n* = 7)	BKV + EBV (*n* = 1)	Nonspecific dysmyelopoiesis (*n* = 38)	TMA (*n* = 3), TB (*n* = 2), MAL (*n* = 1) HS (*n* = 3)

Blood	Parvovirus B19 (*n* = 2)	Parvovirus B19 (*n* = 1) EBV (*n* = 2)	CMV (*n* = 4)	EBV (*n* = 2)	BKV (*n* = 5)	EBV (*n* = 1)	EBV (*n* = 6) CMV (*n* = 2) BKV (*n* = 3) Parvovirus B19 (*n* = 1)	CMV (*n* = 1) EBV (*n* = 2) BKV (*n* = 1)

EBV: Epstein-Barr virus; CMV: cytomegalovirus; TMA: thrombotic microangiopathy; TB: tuberculosis; MAL: myeloid acute leukemia; HS: hemophagocytic syndrome.

**Table 3:** Biological parameters of patients with BKV replication within bone marrow.

Patient number	Time to cytopenia (months)	Hemoglobin level (g/dL)	PMN count (/mm^3^)	Platelet count (/mm^3^)	Lymphocyte count (/mm^3^)	CD4-positive cell count (/mm^3^)	CD8-positive cell count (/mm^3^)	CD19-positive cell count (/mm^3^)	Gamma globulins (g/dL)	eGFR* (mL/min)	Viruses in bone marrow (log_10_⁡copies/mL)	Viruses in blood (log_10_⁡copies/mL)
1	4.5	12.9	713	231,000	700	575	297	58	6.2	54	BKV (3.24)	None
2	20	11.8	800	93,000	373	125	220	35	4.8	19	BKV (3.01)	BKV (3)
3	25	16.5	990	140,000	450	365	390	51	7.8	21	BKV (3.01)-EBV	EBV
4	12	9.4	678	187,000	155	34	27	13	4.5	17	BKV (3.2)	BKV (2.8)
5	12	13.1	430	249,000	500	280	223	100	5.7	19	BKV (3.02)	BKV (5)
6	15	10.7	100	65,000	1000	884	790	0	5	12	BKV (2.74)	BKV (2.8)
7	46.5	10.8	50	115,000	730	—	—	0	6	15	BKV (2.94)	None
8	6.5	13.5	726	99,000	700	297	174	91	—	32	BKV (3.1)	BKV (2.8)

*The glomerular filtration rate was calculated using the Modification of the Diet in Renal Disease (MDRD) equation.

PMN: polymorphonuclear leukocytes; eGFR: estimated glomerular-filtration rate.

**Table 4:** Immunosuppressive therapy given to patients with BKV replication within bone marrow.

Patient no.	Induction therapy	Acute rejection	Acute-rejection treatment	Immunosuppressive therapy regimen	Tac or SRL or CsA levels^§^: (ng/mL)	MPA dose: (mg/kg/d)	Steroid dose: (mg/kg/d)	Virus in bone marrow	Virus in blood
1	Anti-IL2R	Y	Steroid pulses	Belatacept-MPA-S	—	14.3	0.14	BKV	—
2	RATG	N	—	Tac-Lef-S	3 to 6	—	0.07	BKV	BKV
3	RATG	N	—	SRL-MPA-S	8 to 14	11.11	0.11	BKV-EBV	EBV
4	RATG	N	—	Tac-MPA-S	5 to 8	11	0.13	BKV	BKV
5	Anti-IL2R	Y	Steroid pulses RATG	Tac-MPA-S	4 to 7	12.5	0.06	BKV	BKV
6*	Anti-IL2R	Y	Steroid pulses PE Rituximab	Tac-MPA-S	3 to 6	5.5	0.17	BKV	BKV
7	Anti-IL2R	Y	Steroid pulses PE Rituximab	Tac-MPA-S	5 to 8	13.2	0.16	BKV	—
8	Anti-IL2R	N	—	CsA-MPA-S	600 to 900	15.6	0.08	BKV	BKV

*Patient 6 experienced a steroid-sensitive acute rejection, which was treated with steroid pulses. Later, he presented with relapsed membrano-proliferative glomerulonephritis, which required plasma exchanges and rituximab therapy.

^§^Levels correspond to trough levels for tacrolimus and sirolimus, and to the level of cyclosporine A at 2 h after intake.

Abbreviations: Anti-IL2R: anti-interleukin-2 receptors; RATG: rabbit anti-thymocyte globulins; Y: yes, N: no; PE: plasma exchange; MPA: mycophenolic acid; S: steroid; Tac: tacrolimus; Lef: leflunomide; SRL: sirolimus; CsA: cyclosporine A.

**Table 5:** Comparisons between patients with and without BKV replication within bone marrow.

	Patients with BKV replication in bone marrow: *n* = 8	Patients without BKV replication in bone marrow: *n* = 64	*P* value
Male gender (%)	6 (75%)	34 (53%)	ns
Age (years)	61 ± 8	56 ± 12	ns
Serum creatinine (µmol/L)	326 ± 250	197.3 ± 107	0.05
DFG (mL/min)	22.4 ± 15	36.3 ± 21	0.04
Retransplantation (%)	1 (12.5%)	12 (19%)	ns

Immunosuppressive therapy			
RATG (%)	5 (62.5%)	24 (37.5%)	ns
Rituximab (%)	2 (25%)	11 (17%)	ns
Steroid pulses (%)	4 (50%)	20 (31%)	ns
Plasma exchange (%)	2 (25%)	13 (20%)	ns
CNIs at bone-marrow analysis (%)	6 (75%)	57 (89%)	ns
MPA at bone-marrow analysis (%)	7 (87.5%)	53 (83%)	ns
MPA dose at bone-marrow analysis (mg/kg/d)	12 ± 4	18 ± 8	ns
Steroid-sensitive acute-rejection episodes (%)	3 (5%)	10 (15.6%)	ns
Steroid-resistant acute-rejection episodes (%)	1 (12.5%)	8 (12.5%)	ns
Antibody-mediated rejection episodes (%)	2 (25%)	8 (12.5%)	ns
Hemoglobin level at bone-marrow analysis (g/dL)	12.4 ± 2.13	10.85 ± 1.77	0.06
PMN counts at bone-marrow analysis (/mm³)	695 (50–990)	944 (20–8821)	ns
Platelet counts at bone-marrow analysis (/mm³)	147,375 ± 67,692	156,593 ± 102,028	ns
Lymphocyte counts (/mm³)	600 (155–730)	464 (27–3656)	ns
CD4-positive cell counts (/mm³)	294 (34–884)	183 (31–2012)	ns
CD8-positive cell counts (/mm³)	223 (27–790)	161 (12–1293)	ns
CD19-positive cell counts (/mm³)	51 (0–918)	35 (0–239)	ns
Gamma globulin levels (g/L)	5.7 (4.5–7.8)	6.9 (2.7–14)	ns
BK viremia at bone-marrow analysis (%)	5 (62.5%)	4 (6.25%)	0.0004
Cytomegalovirus viremia (%)	0 (0%)	9 (14%)	ns

RATG: rabbit anti-thymocyte globulins; CNIs: calcineurin inhibitors; MPA: mycophenolic acid; PMN: polymorphonuclear leukocytes.

## References

[1] Vanrenterghem Y (2009). Anemia after kidney transplantation. *Transplantation*.

[2] Rerolle J-P, Szelag J-C, Le Meur Y (2007). Unexpected rate of severe leucopenia with the association of mycophenolate mofetil and valganciclovir in kidney transplant recipients. *Nephrology Dialysis Transplantation*.

[3] Zafrani L, Truffaut L, Kreis H (2009). Incidence, risk factors and clinical consequences of neutropenia following kidney transplantation: A Retrospective Study. *American Journal of Transplantation*.

[4] Waldman M, Kopp JB (2007). Parvovirus B19 and the kidney. *Clinical Journal of the American Society of Nephrology*.

[5] Almeida-Porada GD, Ascensao JL (1996). Cytomegalovirus as a cause of pancytopenia. *Leukemia and Lymphoma*.

[6] Knoll GA, Macdonald I, Khan A, Van Walraven C (2003). Mycophenolate mofetil dose reduction and the risk of acute rejection after renal transplantation. *Journal of the American Society of Nephrology*.

[7] Hirsch HH, Steiger J (2003). Polyomavirus BK. *Lancet Infectious Diseases*.

[8] Hirsch HH, Randhawa P (2013). BK polyomavirus in solid organ transplantation. *American Journal of Transplantation*.

[9] Petrogiannis-Haliotis T, Sakoulas G, Kirby J (2001). BK-related polyomavirus vasculopathy in a renal-transplant recipient. *The New England Journal of Medicine*.

[10] Esposito L, Hirsch H, Basse G, Fillola G, Kamar N, Rostaing L (2007). BK virus-related hemophagocytic syndrome in a renal transplant patient. *Transplantation*.

[11] Gardeniers SHM, Mekahli D, Levtchenko E, Lerut E, Renard M, Van Damme-Lombaerts R (2010). Bone marrow aplasia and graft loss in a pediatric renal transplant patient with polyomavirus nephropathy. *Pediatric Nephrology*.

[12] Mengelle C, Sandres-Sauné K, Pasquier C (2003). Automated extraction and quantification of human cytomegalovirus DNA in whole blood by real-time PCR assay. *Journal of Clinical Microbiology*.

[13] Limaye AP, Jerome KR, Kuhr CS (2001). Quantitation of BK virus load in serum for the diagnosis of BK virus—associated nephropathy in renal transplant recipients. *Journal of Infectious Diseases*.

[14] Öhrmalm L, Wong M, Aust C (2012). Viral findings in adult hematological patients with neutropenia. *PLoS ONE*.

